# Adverse events of Capmatinib: A real-world drug safety surveillance study based on the FDA adverse event reporting system (FAERS) database

**DOI:** 10.1097/MD.0000000000041460

**Published:** 2025-01-31

**Authors:** Hao Zhang, Panli Zhao, Hua Huang

**Affiliations:** a Department of Pharmacy, Chengdu Seventh People’s Hospital (Affiliated Tumor Hospital of Chengdu Medical College), Chengdu, Sichuan, China.

**Keywords:** adverse events, Capmatinib, FAERS, tumor

## Abstract

The present study aims to evaluate the adverse events associated with Capmatinib using real-world data, providing a reference basis for its rational use in clinical practice. Relevant data from the Food and Drug Administration adverse event reporting system database was mined. Next, reporting odds ratio and Bayesian confidence propagation neural network method were used to analyze real-world adverse events associated with Capmatinib. The study revealed significant adverse event signals of Capmatinib, primarily involving general disorders and administration site conditions, cardiac disorders, gastrointestinal disorders, respiratory, thoracic and mediastinal disorders, neoplasms benign, malignant and unspecified (including cysts and polyps) and investigations, among others. A total of 79 signals were identified, with 13 of them not mentioned in the drug’s specifications. Taken together, our comprehensive analysis of the Food and Drug Administration adverse event reporting system database enhances the understanding of Capmatinib’s safety profile, thereby contributing to informed decision-making in its clinical application and facilitating the timely management of associated adverse reactions.

## 1. Introduction

Non-small cell lung cancer (NSCLC) stands as the predominant subtype of lung cancer, with around 3% to 4% of patients exhibiting mesenchymal–epithelial transition (MET) exon 14 skipping mutations.^[1]^ Of note, Capmatinib, developed by Novartis Pharmaceuticals AG, received Food and Drug Administration (FDA) approval on May 6, 2020, under the trade name Tabrecta in the united states, to treat adult patients with advanced or metastatic NSCLC harboring the MET exon 14 skipping mutation.^[2]^ Functioning as a selective inhibitor of the MET receptor,^[3]^ Capmatinib targets the MET tyrosine kinase receptor, selectively binding to MET and inhibiting MET phosphorylation. This, in turn, disrupts oncogenic MET receptor signaling induced by alterations in the MET gene, including MET exon 14 knockout mutations and MET protein overexpression, resulting in the death of tumor cells overexpressing MET protein or expressing constitutively activated MET protein.^[4–6]^ Although several adverse effects of Capmatinib have been reported following clinical application, there is still a lack of comprehensive studies based on large databases. To that end, we analyzed adverse events associated with Capmatinib using real-world data obtained from the adverse event reporting system of the U.S. FDA, aiming to establish a reference for the clinical use of this drug.

## 2. Materials and methods

### 2.1. Data sources

Open Vigil 2.1 is an online tool for data mining and pharmacovigilance data analysis that has been widely used in pharmacovigilance studies.^[7]^ Our search spanned from June 1, 2020 to June 1, 2023, and the target drug name used was “Capmatinib” to obtain adverse drug event (ADE) data.

### 2.2. Data standardization

The preferred terminology (PT) for ADE descriptors was standardized and simplified with reference to the Medical Dictionary of Regulatory Activities. ADE reports belonging to the same PT were combined, and the PTs were categorized according to the system organ classification (SOC).

### 2.3. Signal detection methods

The ratio imbalance method (proportional disproportionality metric) is a widely used ADR signal detection method in world.^[8]^ Herein, the reported ratio of ratios (ROR) method was employed in conjunction with the Bayesian confidence interval progressive neural network (BCPNN) method for data mining. The detailed calculation formulas can be found in Tables 1 and 2. Each PT was judged and screened according to the thresholds specified in Table 2, and the judgmental criteria of the 2 algorithms had to be satisfied simultaneously in order to generate 1 ADE signal. The generation of ADE signals indicates a statistical correlation between ADE and the drug being assessed. The stronger the signal, the stronger the correlation between ADE signals and drugs.

**Table 1:** Fourfold table matrix.

	Topotecan	Non-topotecan
Target AEs	a	c
Nontarget AEs	b	d
N = a + b + c + d		

AEs = adverse drug events.

**Table 2:** Formulas and thresholds of ROR and BCPNN.

Method	Formula	Threshold
ROR	ROR=$\frac{a/c}{b/d}$ 95% CI = e^($\ln ROR\pm 1.96\sqrt{1/a+1/b+1/c+1/d}$)	*a* ≥ 3% and 95% CI (lower limit) > 1
BCPNN	IC=$\log_{2}\frac{a\left(a+b+c+d\right)}{\left(a+b\right)\left(a+c\right)}$ $\gamma =\gamma_{ij}\frac{\left(N+\alpha \right)\left(N+\beta \right)}{\left(a+b+\alpha_{i}\right)\left(a+c+\beta_{j}\right)}$ $E\left(IC\right)=\log_{2}\frac{\left(a+\gamma_{ij}\right)\left(N+\alpha \right)\left(N+\beta \right)}{\left(N+\gamma \right)\left(a+b+\alpha_{i}\right)\left(a+c+\beta_{j}\right)}$ $V\left(IC\right)={\left(\frac{1}{\ln2}\right)}^{2}\left(\frac{N-\alpha +\gamma -\gamma_{ij}}{\left(\alpha +\gamma_{ij}\right)\left(1+N+\gamma \right)}+\frac{N-a-b+\alpha -\alpha_{i}}{\left(a+b+\alpha_{i}\right)\left(1+N+\alpha \right)}+\frac{N-a-c+\beta -\beta_{j}}{\left(a+c+\beta_{j}\right)\left(1+N+\beta \right)}\right)$ $SD=\sqrt{V\left(IC\right)}$	
	IC025=E(IC)−2SD	IC025 > 0

BCPNN = Bayesian confidence propagation neural network, CI = confidence interval, IC = information coefficient, ROR = reporting odds ratio.

## 3. Results

### 3.1. ADE signals associated with Capmatinib

From June 1, 2020 to June 1, 2023, a total of 4335 adverse drug event reports involving Capmatinib as the primary suspect drug were collected. Following the criteria outlined for ADE signal determination, 79 signals related to Capmatinib were identified. These signals encompassed 2201 ADE reports, which are detailed in Table 3.

**Table 3:** Basic information of Capmatinib ADE signal.

Basic information	Categorization	Number of reports	Composition ratio (%)
Gender	Male	262	11.90%
	Female	285	12.95%
	Unknown or missing	1654	75.15%
Age groups	18–49	3	0.14%
	50–69	155	7.04%
	≥70	386	17.54%
	Unknown or missing	1657	75.28%
Country or area	United States	216	9.81%
	Japan	48	2.18%
	Europe	126	5.72%
	Other	1811	82.28%

ADE = adverse drug event.

### 3.2. SOC classification

**Figure 1.**
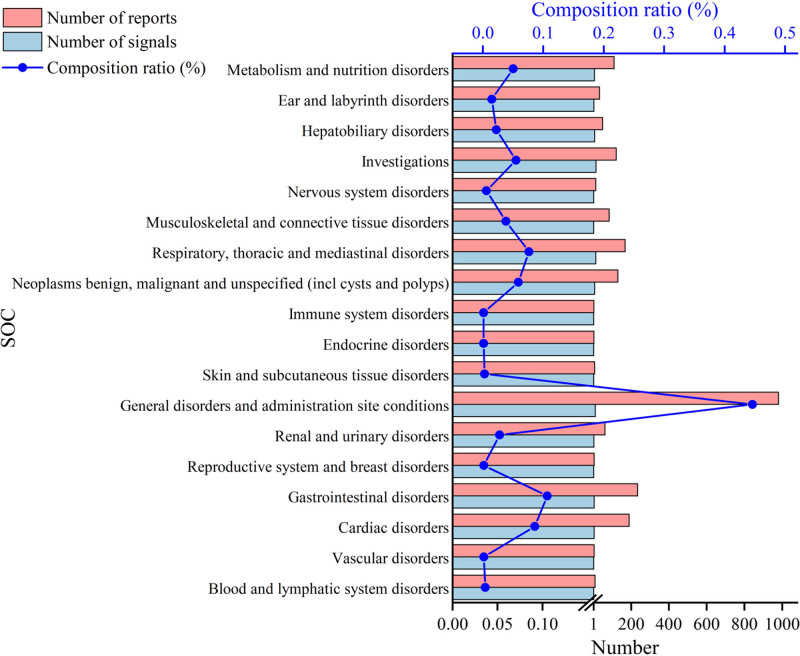
SOC classification of ADE signals. ADE = adverse drug event, SOC = system organ classification.

The 79 adverse drug event signals associated with Capmatinib spanned across 18 SOCs. These mainly included general disorders and administration site conditions, cardiac disorders, gastrointestinal disorders, respiratory, thoracic and mediastinal disorders, neoplasms benign, malignant and unspecified (including cysts and polyps) and investigations (SOC classification), among others. The specific information is shown in Figure 1.

### 3.3. ADE risk signals

**Figure 2.**
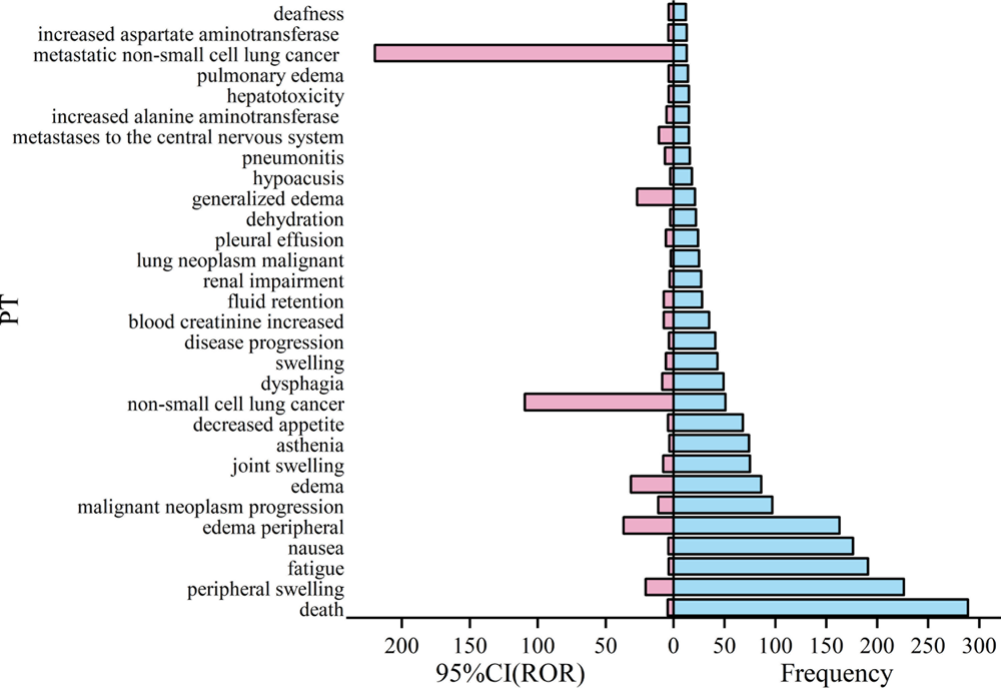
Sorted by frequency of occurrence.

**Figure 3.**
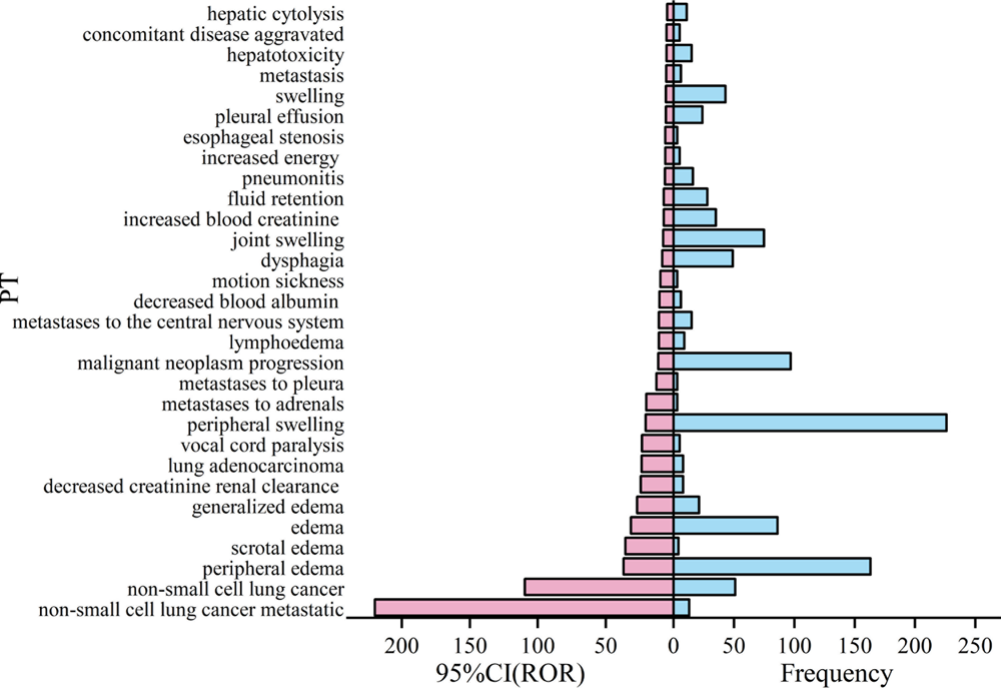
Sorted by ROR signal strength, ROR = reporting odds ratio.

Next, the 79 ADE signals were ranked according to frequency and signal intensity [lower limit of 95% CI (ROR)], and the top 30 PTs are shown in Figures 2 and 3. Noteworthy observations include PTs with higher frequency, such as death, peripheral swelling, fatigue, nausea, and peripheral edema. The PTs with a higher signal intensity include death, peripheral swelling, scrotal edema, edema, generalized edema, and decreased renal creatinine clearance, peripheral edema, scrotal edema, edema, and generalized edema.

### 3.4. Newly discovered suspected adverse reactions

As shown in Table 4, the 79 ADE signals were graded based on signal strength to exclude signals unrelated to the adverse reactions of the drug itself, such as production issues, injuries, poisoning, surgical complications, pregnancy, puerperium and perinatal conditions, and various congenital familial hereditary diseases. Following this exclusion process and a thorough comparison with the drug prescribing information, a total of 79 signals were identified. Among these, 66 were explicitly mentioned or related within the instruction manual, while 13 were not stated. Detailed information on these suspected adverse reactions is presented in Table 4.

**Table 4:** Suspected adverse reaction of Capmatinib.

SOC	PT (case numbers)
Blood and lymphatic system disorders	Lymphedema (9)
Vascular disorders	Pulmonary thrombosis (4)*
Cardiac disorders	Peripheral swelling (163) pulmonary edema (14) hemoptysis (7)* ascites (5)
Gastrointestinal disorders	Dysphagia (49)* esophageal stenosis (3)* nausea (176) retching (6)
Reproductive system and breast disorders	Scrotal edema (4)
Renal and urinary disorders	Fluid retention (28) renal impairment (27) chromaturia (6)
General disorders and administration site conditions	Edema (86) generalized edema (21) peripheral edema (226) increased energy (5)* swelling (43) concomitant disease aggravation (5) death (289) fatigue (191) disease progression (41) asthenia (74)
Skin and subcutaneous tissue disorders	Photosensitivity reaction (6)
Endocrine disorders	Metastases to the adrenal glands (3)
Immune system disorders	Swelling of eyelid (3)
Neoplasms benign, malignant and unspecified (including cysts and polyps)	Metastases to the pleura (3) malignant neoplasm progression (97) metastases to central nervous system (15) metastasis (6) neoplasm (5) brain neoplasm (3)
Respiratory, thoracic and mediastinal disorders	Metastatic non-small cell lung cancer (13) non-small cell lung cancer (51) lung adenocarcinoma (8) vocal cord paralysis (5) pneumonitis (16) pleural effusion (24) malignant lung neoplasm (25) lung carcinoma cell type unspecified stage iv (5) choking (5)* pulmonary mass (4) metastases to the lungs (3) exertional dyspnea (6) pulmonary fibrosis (3)
Musculoskeletal and connective tissue disorders	Joint swelling (75) metastases to the bones (9)
Nervous system disorders	Central nervous system lesion (5) taste disorder (8)*
Investigations	Decreased creatinine renal clearance (86) decreased blood albumin (3) increased blood creatinine (5) increased amylase levels (3) increased liver function test (11) increased aspartate aminotransferase (13) increased gamma-glutamyl transferase (6) increased alanine aminotransferase (15) decreased blood sodium (6) increased blood bilirubin (5) decreased blood iron (4)* decreased glomerular filtration rate (3) increased blood alkaline phosphatase (3) abnormal liver function test (3)
Hepatobiliary disorders	Hepatotoxicity (15) hepatic cytolysis (11) hypoalbuminemia (3) hyperbilirubinemia (3) cholestasis (4) hepatitis (5) liver disorder (8)
Ear and labyrinth disorders	Motion sickness (3) deafness (12)* hypoacusis (18)*
Metabolism and nutrition disorders	Increased appetite (8)* decreased appetite (68) dehydration (22)* hyponatremia (8) eating disorder (4)*

PT = preferred terminology, SOC = system organ classification.

## 4. Discussion

### 4.1. Analysis of ADE signals

The male-to-female ratio in the reported cases was close to 1:1, with a slightly higher prevalence in women. Most patients were elderly at disease onset, consistent with the epidemiological patterns.^[8]^ The primary source of reports was from the United States, followed by Europe, possibly influenced by regional variations in drug usage frequencies and disparities in disease incidence among different races.^[9]^ The presence of missing data underscores the importance of meticulous reporting by medical staff to accurately reflect patient information in medical records, facilitating comprehensive data analysis.

### 4.2. SOC classification of ADE signals

Among the 18 SOCs accrued by ADE signals, the highest number of reports included general disorders and administration site conditions, cardiac disorders, gastrointestinal disorders, respiratory, thoracic and mediastinal disorders, neoplasms benign, malignant and unspecified (including cysts and polyps) and investigations, among others,^[10]^ aligning with the drug prescribing information. Notably, General disorders and administration site conditions ranked first and were significantly more prevalent than other SOCs, emphasizing the need for heightened clinical vigilance and effective patient communication concerning these adverse reactions.

### 4.3. ADE signal analysis

Analysis of the combined frequency and signal intensity of ADEs associated with Capmatinib revealed peripheral edema, nausea, fatigue, vomiting, elevated blood pressure, and elevated blood glucose as the most common reactions during treatment.^[11]^ Moreover, monitoring for new or worsening pulmonary symptoms indicative of interstitial lung disease or pneumonia is advised.^[12]^ Regular liver function tests are also recommended to prevent hepatotoxicity, with treatment adjustments based on severity.^[13,14]^ Additional warnings include embryo-fetal toxicity, necessitating evaluation of women of childbearing potential throughout treatment. Furthermore, Capmatinib may increase photosensitivity, requiring patients to limit direct UV exposure.^[15]^

### 4.4. Newly identified suspected adverse reactions

There were 13 newly identified adverse reactions in this study, encompassing ear and labyrinthine, metabolic and nutritional, neurologic, cardiac, respiratory, thoracic, and mediastinal disorders. This might be associated with metastases affecting the central nervous system, which are common in late-stage NSCLC patients,^[16]^ or a result of Capmatinib impairing the transmission of taste signals through the neural pathway, resulting in loss of taste function, abnormal taste, and hyposmia. In addition, hearing loss was reported in 18 cases, and deafness in 12 cases. A previous study reported brain metastases in 3.8% of NSCLC cases, particularly adenocarcinomas, with nearly one-third of patients exhibiting central nervous system metastases alongside brain metastases.^[17]^ As hearing is transmitted through nerves and ultimately reaches the hearing center, sudden hearing loss in patients warrants prompt consideration of potential brain metastasis or central nervous system metastasis. As an added precaution, a reduction in the drug dose is also recommended. Furthermore, instances of hemoptysis and pulmonary thrombosis were also observed. These clinical manifestations underscore the importance of close monitoring and the need for timely as well as appropriate treatment to alleviate patient distress and minimize associated pain.

### 4.5. Limitations

While this study provides valuable insights, several limitations and considerations should be acknowledged The FDA adverse event reporting system database is a self-reporting system, introducing challenges such as data omission, misreporting, and information gaps. The information comes from a variety of sources (e.g., pharmaceutical companies, patients, doctors, etc), leading to a certain degree of reporting bias.^[18]^ Despite employing the combined ROR and Bayesian confidence interval progressive neural network methods to improve the screening threshold of ADE signals, the possibility of false-positive ADE signals cannot be fully excluded. ADE signals generated from the data can only establish a statistical correlation between the drug and adverse events. Causality remains a subject for further clinical research and evaluation. More in-depth studies are required to discern the true cause-and-effect relationships. The dataset predominantly originates from Europe and the United States. The applicability of findings to the Chinese patient population or other populations requires further validation.

## Author contributions

**Data curation:** Panli Zhao.

**Writing – original draft:** Hao Zhang.

**Writing – review & editing:** Hua Huang.

## Supplementary Material
