# Supplementary material for: Adverse events of Capmatinib: A real-world drug safety surveillance study based on the FDA adverse event reporting system (FAERS) database
Source: Medicine (Baltimore). 2025 Jan 31;104(5):e41460. doi: 10.1097/MD.0000000000041460 (PMC11789861; doi:10.1097/MD.0000000000041460)
Supplement: Supplementary file 1 [file medi-104-e41460-s001.docx]

$$R^{2}=\frac{2\times\beta^{2}\times EAF\times\left(1-EAF\right)}{2\times\beta^{2}\times EAF\times\left(1-EAF\right)+2\times SE^{2}\times N\times EAF\times\left(1-EAF\right)}$$

$$\text{F}=\frac{N-K-1}{K}\times\frac{R^{2}}{1-R^{2}}$$

N, the sample size of the GWASs; K, the number of the SNPs; R^2^, the instrumental variable explains the degree of exposure (determinant coefficient of regression equation); EAF, effect allele frequency for the genetic variant of interest; β, the effect size for the genetic variant of interest; SE, standard error of effect size for the genetic variant of interest.

Supplementary Table 1

| **Table S2. Trait associated with all SNPs obtained in PheWAS** | | | | | | | | |
| --- | --- | --- | --- | --- | --- | --- | --- | --- |
|  |  |  |  |  |  |  |  |  |
| SNP | GWAS ID | Trait | Position | *P* | SE | N | Beta | EAF |
| rs10515808 | ieu-a-1110 | Large vessel disease | 159820931 | 0.000126 | 0.0559 | 21143 | 0.2144 | 0.1031 |
|  | ukb-e-102060_AFR | Danish pastry intake | 159820931 | 0.000145902 | 0.04632 | NA | -0.1759 | 0.97098 |
|  | finn-b-VD_MI | Vascular dementia (multiple infarctations) | 159820931 | 0.000296203 | 0.1901 | NA | 0.688 | 0.08472 |
|  | ebi-a-GCST006907 | Ischemic stroke (large artery atherosclerosis) | 159820931 | 0.000308702 | 0.0411 | 410484 | 0.1483 | 0.1052 |
|  | ebi-a-GCST006061 | Atrial fibrillation | 159820931 | 0.0003596 | 0.0124 | 537409 | 0.0444 | NA |
|  | ebi-a-GCST90002240 | Fasting insulin | 159820931 | 0.000393704 | 0.0222 | 8353 | 0.0844 | 0.112 |
|  | ieu-b-32 | lymphocyte cell count | 159820931 | 0.000429003 | 0.003171 | 523522 | 0.011174 | 0.098619 |
|  | finn-b-J10_PNEUMONONBACT | Pneumonia due to other infectious organisms, not elsewhere classified | 159820931 | 0.000432504 | 0.1326 | NA | 0.4666 | 0.08514 |
|  | ukb-e-6179_p5_CSA | Mineral and other dietary supplements | 159820931 | 0.000436697 | 0.1529 | NA | -0.5378 | 0.92726 |
|  | finn-b-M13_TOEDEFORMOTH | Other deformities of toe(s) | 159820931 | 0.000600703 | 0.1168 | NA | -0.4009 | 0.08475 |
|  | ukb-a-256 | Other eye problems | 159820931 | 0.000730685 | 0.00142947 | 336428 | 0.00482852 | 0.0984686 |
|  | finn-b-J10_HYPERSPNEUMONI | Hypersensitivity pneumonitis due to organic dust | 159820931 | 0.000891292 | 0.1654 | NA | -0.5496 | 0.08478 |
| rs17702901 | ebi-a-GCST004458 | Granulocyte-colony stimulating factor levels | 92930411 | 0.000169602 | 0.0396 | 7904 | -0.147 | NA |
|  | ukb-e-6155_p2_CSA | Vitamin and mineral supplements | 92930411 | 0.000200198 | 0.05664 | NA | -0.2106 | 0.90403 |
|  | ebi-a-GCST90016620 | Schizophrenia vs autism spectrum disorder (ordinary least squares (OLS)) | 92930411 | 0.000309999 | 0.003 | 59056 | 0.011 | NA |
|  | ukb-e-4100_CSA | Ankle spacing width (left) | 92930411 | 0.000385097 | 0.03052 | NA | -0.1083 | 0.8961 |
|  | bbj-a-7 | Activated partial thromboplastin time | 92930411 | 0.000528506 | 0.01006 | NA | -0.03486 | 0.1558 |
|  | met-d-L_LDL_TG_pct | Triglycerides to total lipids ratio in large LDL | 92930411 | 0.000539995 | 0.0109714 | NA | 0.0385752 | 0.036018 |
|  | prot-a-659 | Corticotropin-releasing factor-binding protein | 92930411 | 0.000549541 | 0.0675 | 3301 | 0.2335 | 0.03669 |
|  | met-d-IDL_TG_pct | Triglycerides to total lipids ratio in IDL | 92930411 | 0.000549997 | 0.0108204 | NA | 0.0388857 | 0.036018 |
|  | finn-b-M13_ACHILLESTEND | Achilles tendinitis | 92930411 | 0.000592393 | 0.0861 | NA | -0.2957 | 0.0467 |
|  | ukb-b-969 | Time spend outdoors in summer | 92930411 | 0.000629999 | 0.00524036 | 419314 | 0.0179233 | 0.035899 |
|  | ubm-a-3073 | a2009s rh G cingul-Post-dorsal thickness | 92930411 | 0.000645654 | 0.0384 | 7916 | 0.1311 | 0.036992 |
|  | ubm-a-2965 | a2009s lh G&S cingul-Mid-Ant thickness | 92930411 | 0.000676083 | 0.0395 | 7916 | 0.1345 | 0.036992 |
|  | finn-b-I9_HYPERTROCARDMYOP | Hypertrophic cardiomyopathy | 92930411 | 0.000697204 | 0.1443 | NA | 0.4892 | 0.04669 |
|  | ukb-e-20002_p62_CSA | Non-cancer illness code, self-reported | 92930411 | 0.000805508 | 0.1117 | NA | -0.3744 | 0.8966 |
|  | ukb-a-261 | Hearing difficulty/problems with background noise | 92930411 | 0.000854339 | 0.00322141 | 330759 | -0.0107421 | 0.0356881 |
|  | ebi-a-GCST90010121 | Dickkopf-related protein 1 levels | 92930411 | 0.000875871 | 0.189186 | 1301 | -0.634809 | 0.011 |
|  | ebi-a-GCST90010117 | Cathepsin L1 levels | 92930411 | 0.000889836 | 0.189782 | 1301 | -0.63475 | 0.011 |
|  | prot-a-1957 | Growth/differentiation factor 8 | 92930411 | 0.000891251 | 0.0675 | 3301 | -0.2245 | 0.03669 |
|  | ebi-a-GCST005217 | Insulin levels adjusted for BMI | 92930411 | 0.000937001 | 0.052 | 4409 | 0.17 | 0.95 |
|  | ukb-e-24003_CSA | Nitrogen dioxide air pollution; 2010 | 92930411 | 0.000987302 | 0.02152 | NA | -0.07089 | 0.8949 |
| rs7129556 | ukb-a-374 | Hand grip strength (left) | 77300048 | 3.84017E-07 | 0.00197989 | 335821 | -0.0100516 | 0.266896 |
|  | ukb-b-10215 | Hand grip strength (right) | 77300048 | 0.0000051 | 0.0016796 | 461089 | -0.00765934 | 0.26724 |
|  | ukb-a-379 | Hand grip strength (right) | 77300048 | 5.62549E-06 | 0.00198386 | 335842 | -0.00900688 | 0.266896 |
|  | ukb-b-7478 | Hand grip strength (left) | 77300048 | 0.00004 | 0.0016768 | 461026 | -0.00688872 | 0.267231 |
|  | ukb-b-2966 | Milk intake | 77300048 | 0.00012 | 0.0028202 | 64943 | -0.010864 | 0.267234 |
|  | ukb-d-30110_irnt | Platelet distribution width | 77300048 | 0.000142515 | 0.00266944 | 350470 | -0.010154 | 0.267277 |
|  | ukb-e-104370_AFR | Watercress intake | 77300048 | 0.0001986 | 0.01825 | NA | -0.06792 | 0.8474 |
|  | finn-b-R18_DISTU_SKIN_SENSA | Disturbances of skin sensation | 77300048 | 0.000320501 | 0.0283 | NA | -0.1018 | 0.2169 |
|  | finn-b-C3_FEMALE_GENITAL_NAS | Malignant neoplasm of other and unspecified female genital organs | 77300048 | 0.000485199 | 0.1694 | NA | 0.5911 | 0.2173 |
|  | ukb-d-I9_STR_SAH | Stroke, including SAH | 77300048 | 0.000551087 | 0.000293894 | 361194 | -0.0010153 | 0.2673 |
|  | finn-b-O15_LABOUR_OTHER | Other complications of labour and delivery, not elsewhere classified | 77300048 | 0.000552103 | 0.0534 | NA | 0.1843 | 0.2167 |
|  | finn-b-C3_FEMALE_GENITAL_NAS_EXALLC | Malignant neoplasm of other and unspecified female genital organs (all cancers excluded) | 77300048 | 0.000557301 | 0.1691 | NA | 0.5838 | 0.2169 |
|  | ukb-b-20145 | Cancer code, self-reported: colon cancer/sigmoid cancer | 77300048 | 0.00061 | 0.000133252 | 462933 | 0.00045688 | 0.267252 |
|  | finn-b-L12_NECROLIPOIDIC | Necrobiosis lipoidica, not elsewhere classified | 77300048 | 0.0006475 | 0.176 | NA | -0.6003 | 0.217 |
|  | ukb-e-48_CSA | Waist circumference | 77300048 | 0.000655707 | 0.0144 | NA | -0.04907 | 0.7731 |
|  | ieu-a-108 | Waist-to-hip ratio | 77300048 | 0.000719996 | 0.0086 | 34519 | -0.029 | 0.2167 |
|  | ukb-b-13616 | Number of baps with butter/margarine | 77300048 | 0.000920005 | 0.00172943 | 64942 | 0.0057295 | 0.267253 |
| rs7158359 | ebi-a-GCST90017075 | Gut microbiota abundance (genus Tyzzerella3 id.11335) | 89594295 | 4.40504E-06 | 0.0249195 | 14306 | -0.114352 | NA |
|  | ukb-b-13344 | Herbal tea intake | 89594295 | 0.00021 | 0.154241 | 64949 | 0.572357 | 0.182131 |
|  | ukb-b-18042 | Illnesses of siblings: Diabetes | 89594295 | 0.00025 | 0.000850591 | 362826 | 0.00311662 | 0.183757 |
|  | ukb-b-18469 | Particulate matter air pollution (pm10); 2010 | 89594295 | 0.000369999 | 0.00281104 | 423796 | 0.0100173 | 0.183253 |
|  | ebi-a-GCST90012069 | vascular endothelial growth factor D levels | 89594295 | 0.000456604 | 0.0151 | 21758 | 0.0529 | 0.1744 |
|  | ukb-d-L12_ATROPHICSKIN | Atrophic disorders of skin | 89594295 | 0.000566879 | 0.000212171 | 361194 | 0.000731356 | 0.183751 |
|  | ukb-d-L90 | Diagnoses - main ICD10: L90 Atrophic disorders of skin | 89594295 | 0.000566879 | 0.000212171 | 361194 | 0.000731356 | 0.183751 |
|  | prot-a-473 | Cyclin-dependent kinase 1:G2/mitotic-specific cyclin-B1 complex | 89594295 | 0.00060256 | 0.0318 | 3301 | -0.1092 | 0.19546 |
|  | ukb-d-L12_SCARCONDITIONS | Scar conditions and fibrosis of skin | 89594295 | 0.000646502 | 0.000194572 | 361194 | 0.000663751 | 0.183751 |
|  | ukb-e-20086_p1_AFR | Type of special diet followed | 89594295 | 0.000814404 | 0.1104 | NA | -0.3695 | 0.6355 |
|  | ukb-a-223 | Illnesses of siblings: Diabetes | 89594295 | 0.000902194 | 0.000971456 | 259921 | 0.00322466 | 0.182908 |
|  | prot-a-1222 | Glycolipid transfer protein | 89594295 | 0.000954993 | 0.0318 | 3301 | 0.105 | 0.19546 |
|  | ukb-e-550_AFR | Abdominal hernia | 89594295 | 0.000999609 | 0.06492 | NA | -0.2136 | 0.5978 |
| rs7185923 | ukb-d-30210_irnt | Eosinophill percentage | 51245487 | 1.68221E-08 | 0.00238754 | 349861 | -0.0134705 | 0.479681 |
|  | ieu-b-33 | eosinophil cell count | 51245487 | 4.25001E-07 | 0.001993 | 473151 | -0.010086 | 0.480751 |
|  | ukb-a-389 | Standing height | 51245487 | 6.23505E-06 | 0.00173195 | 336474 | -0.00782553 | 0.479115 |
|  | ukb-a-234 | Forced expiratory volume in 1-second (FEV1) predicted | 51245487 | 0.000017258 | 0.00273394 | 110423 | -0.0117502 | 0.479115 |
|  | ieu-a-89 | Height | 51245487 | 1.89998E-05 | 0.003 | 251065 | -0.013 | 0.467 |
|  | ukb-b-8428 | Forced expiratory volume in 1-second (FEV1), predicted | 51245487 | 2.69998E-05 | 0.00233152 | 148653 | -0.00979169 | 0.480149 |
|  | ukb-b-10787 | Standing height | 51245487 | 2.69998E-05 | 0.00131585 | 461950 | -0.0055199 | 0.480096 |
|  | finn-b-K11_DISORD_TEETH | Other disorders of teeth and supporting structures | 51245487 | 5.47003E-05 | 0.026 | NA | 0.1048 | 0.5569 |
|  | ukb-b-7012 | Operative procedures - secondary OPCS: Z67.6 Lumbosacral joint | 51245487 | 0.000109999 | 0.000105597 | 463010 | -0.000407688 | 0.480126 |
|  | ukb-a-35 | Comparative height size at age 10 | 51245487 | 0.000146828 | 0.00166347 | 332021 | -0.00631524 | 0.479115 |
|  | finn-b-J10_UPPERDIS | Other diseases of upper respiratory tract | 51245487 | 0.000280098 | 0.0079 | NA | -0.0286 | 0.5565 |
|  | ebi-a-GCST90001761 | CD20 on switched memory B cell | 51245487 | 0.000319499 | 0.02486 | 3657 | -0.08954 | 0.4614 |
|  | ieu-b-35 | C-Reactive protein level | 51245487 | 0.000451004 | 0.003733 | 204402 | 0.013104 | 0.491342 |
|  | ukb-d-30710_irnt | C-reactive protein | 51245487 | 0.000484741 | 0.0023977 | 343524 | 0.0083658 | 0.4797 |
|  | ebi-a-GCST004452 | Interleukin-5 levels | 51245487 | 0.000488203 | 0.0249 | 3364 | -0.0868 | NA |
|  | ebi-a-GCST90001757 | CD20 on IgD- CD38dim B cell | 51245487 | 0.000502805 | 0.02468 | 3657 | -0.08593 | 0.4614 |
|  | prot-a-1392 | Serine protease HTRA2, mitochondrial | 51245487 | 0.000549541 | 0.0248 | 3301 | 0.0856 | 0.47101 |
|  | prot-a-421 | Antigen-presenting glycoprotein CD1d | 51245487 | 0.000645654 | 0.0248 | 3301 | -0.0846 | 0.47101 |
|  | ukb-a-285 | Arm predicted mass (right) | 51245487 | 0.000661394 | 0.00151342 | 331216 | -0.00515339 | 0.479115 |
|  | finn-b-M13_PATELLARTEND | Patellar tendinitis | 51245487 | 0.000724403 | 0.0982 | NA | -0.3318 | 0.5561 |
|  | ukb-a-292 | Trunk fat-free mass | 51245487 | 0.000746122 | 0.00153535 | 331030 | -0.00517731 | 0.479115 |
|  | ebi-a-GCST003724 | Bipolar disorder | 51245487 | 0.000781196 | 0.0228 | 34950 | 0.0803 | 0.4802 |
|  | ukb-b-15942 | Operative procedures - main OPCS: W87.9 Unspecified diagnostic endoscopic examination of knee joint | 51245487 | 0.000809991 | 0.000163637 | 463010 | -0.000548304 | 0.480126 |
|  | prot-a-1580 | T-cell immunomodulatory protein | 51245487 | 0.000870964 | 0.0248 | 3301 | -0.0825 | 0.47101 |
|  | ukb-b-1917 | Operation code: hand/finger surgery | 51245487 | 0.000909997 | 0.000137896 | 462933 | 0.000457295 | 0.480129 |
|  | ukb-e-30130_MID | Monocyte count | 51245487 | 0.000924294 | 0.03767 | NA | -0.1248 | 0.5772 |
|  | ukb-e-5292_CSA | 3mm index of best keratometry results (left) | 51245487 | 0.000956709 | 0.01544 | NA | -0.051 | 0.3894 |
|  | ukb-e-46_EAS | Hand grip strength (left) | 51245487 | 0.000966296 | 0.01812 | NA | -0.05979 | 0.4326 |
| *SE, standard error；EAF, effect allele frequency;* | | | | | | | | |

**Table S3.** The preliminary results of mendelian randomization analyses

| Outcomes | N | IVW | | WM | | ME | |
| --- | --- | --- | --- | --- | --- | --- | --- |
|  |  | OR (95% CI) | *p* | OR (95% CI) | *p* | OR (95% CI) | *p* |
| Breast cancer | 4 | 0.820(0.815−0.825) | 0.00e+00 | 0.868(0.836-0.901) | 1.72e-13 | 0.971(0.884- 1.067) | 0.603 |
| Lung cancer | 4 | 0.992(0.987-0.997) | 0.002 | 0.990(0.984-0.997) | 0.003 | 0.984(0.972-0.997) | 0.051 |
| Cervical cancer | 3 | 1.000123(1.0000313-1.000215) | 0.009 | 1.000116 (0.9999857- 1.000246) | 0.081 | 1.000223(0.9998791- 1.000566) | 0.425 |
| Haemotological cancer | 3 | 0.9998462(0.9997088-0.9999836) | 0.028 | 0.9998886(0.9997090-1.0000683) | 0.224 | 0.9998269(0.9994254-1.0002285) | 0.553 |
| Thyroid cancer | 4 | 0.994(0.972-1.0116) | 0.591 | 0.984(0.957-1.012) | 0.268 | 0.970(0.899-1.047) | 0.515 |
| Gastric cancer | 4 | 0.995(0.968-1.022) | 0.705 | 0.988(0.956-1.021) | 0.463 | 0.964(0.910-1.022) | 0.346 |
| Pancreatic cancer | 4 | 0.978(0.951-1.006) | 0.117 | 0.980(0.950-1.011) | 0.202 | 0.980(0.922-1.041) | 0.580 |
| Bladder cancer | 3 | 0.9999767(0.9999030-1.000051) | 0.537 | 0.9999707(0.9998740-1.000068) | 0.553 | 1.0000146(0.9998103-1.000219) | 0.9111558 |
| Melanoma skin cancer | 3 | 1.0000154(0.9998836-1.000147) | 0.809 | 1.0000562(0.9998992-1.000213) | 0.483 | 0.9999984(0.9996001-1.000397) | 0.995 |
| Brain cancer | 3 | 0.9999941(0.9999432-1.000045) | 0.821 | 0.9999992(0.9999383-1.000060) | 0.979 | 0.9999561(0.9998468-1.000065) | 0.576 |
| Colorectal cancer | 3 | 1.000146(0.9999938-1.000299) | 0.060 | 1.000186(1.0000045-1.000367) | 0.045 | 1.000126(0.9996771-1.000575) | 0.680 |
| Liver & bile duct cancer | 3 | 1.000018(0.9999795-1.000057) | 0.356 | 1.000023(0.9999780- 1.000069) | 0.314 | 1.000025(0.9999414-1.000108) | 0.666 |
| Prostate cancer | 4 | 1.000068(0.9999198-1.000216) | 0.369 | 1.000118(0.9999146-1.000322) | 0.256 | 1.000495(1.0001438-1.000847) | 0.110 |
| Ovarian cancer | 3 | 1.0000302(0.9998955-1.000165) | 0.661 | 0.9999897(0.9998068-1.000173) | 0.912 | 0.9997121(0.9994227-1.000002) | 0.302 |
| Endometrial cancer | 4 | 1.000329(0.9925985-1.008120) | 0.934 | 1.002520(0.9924106-1.012733) | 0.626 | 1.006137(0.9878408-1.024771) | 0.581 |
| Head and neck cancer | 3 | 1.0000274(0.9999587-1.000096) | 0.435 | 1.0000383(0.9999576-1.000119) | 0.352 | 0.9999484(0.9998010-1.000096) | 0.617 |
| Oesophageal cancer | 3 | 1.000052(0.9999959-1.000108) | 0.069 | 1.000053(0.9999896-1.000116) | 0.102 | 1.000027(0.9999066-1.000148) | 0.734 |
| Oral cavity cancer | 3 | 1.0000078(0.9999687-1.000047) | 0.694 | 1.0000013(0.9999550-1.000048) | 0.956 | 0.9999721(0.9998881-1.000056) | 0.633 |

OR, odds ratio; CI, Confidence interval.

**Table S3.** The preliminary results of mendelian randomization analyses

| Outcomes | N | IVW | | WM | | ME | |
| --- | --- | --- | --- | --- | --- | --- | --- |
|  |  | OR (95% CI) | *p* | OR (95% CI) | *p* | OR (95% CI) | *p* |
| Breast cancer | 4 | 0.820(0.815−0.825) | 0.00e+00 | 0.868(0.836-0.901) | 1.72e-13 | 0.971(0.884- 1.067) | 0.603 |
| Lung cancer | 4 | 0.992(0.987-0.997) | 0.002 | 0.990(0.984-0.997) | 0.003 | 0.984(0.972-0.997) | 0.051 |
| Cervical cancer | 3 | 1.000123(1.0000313-1.000215) | 0.009 | 1.000116 (0.9999857- 1.000246) | 0.081 | 1.000223(0.9998791- 1.000566) | 0.425 |
| Haemotological cancer | 3 | 0.9998462(0.9997088-0.9999836) | 0.028 | 0.9998886(0.9997090-1.0000683) | 0.224 | 0.9998269(0.9994254-1.0002285) | 0.553 |
| Thyroid cancer | 4 | 0.994(0.972-1.0116) | 0.591 | 0.984(0.957-1.012) | 0.268 | 0.970(0.899-1.047) | 0.515 |
| Gastric cancer | 4 | 0.995(0.968-1.022) | 0.705 | 0.988(0.956-1.021) | 0.463 | 0.964(0.910-1.022) | 0.346 |
| Pancreatic cancer | 4 | 0.978(0.951-1.006) | 0.117 | 0.980(0.950-1.011) | 0.202 | 0.980(0.922-1.041) | 0.580 |
| Bladder cancer | 3 | 0.9999767(0.9999030-1.000051) | 0.537 | 0.9999707(0.9998740-1.000068) | 0.553 | 1.0000146(0.9998103-1.000219) | 0.9111558 |
| Melanoma skin cancer | 3 | 1.0000154(0.9998836-1.000147) | 0.809 | 1.0000562(0.9998992-1.000213) | 0.483 | 0.9999984(0.9996001-1.000397) | 0.995 |
| Brain cancer | 3 | 0.9999941(0.9999432-1.000045) | 0.821 | 0.9999992(0.9999383-1.000060) | 0.979 | 0.9999561(0.9998468-1.000065) | 0.576 |
| Colorectal cancer | 3 | 1.000146(0.9999938-1.000299) | 0.060 | 1.000186(1.0000045-1.000367) | 0.045 | 1.000126(0.9996771-1.000575) | 0.680 |
| Liver & bile duct cancer | 3 | 1.000018(0.9999795-1.000057) | 0.356 | 1.000023(0.9999780- 1.000069) | 0.314 | 1.000025(0.9999414-1.000108) | 0.666 |
| Prostate cancer | 4 | 1.000068(0.9999198-1.000216) | 0.369 | 1.000118(0.9999146-1.000322) | 0.256 | 1.000495(1.0001438-1.000847) | 0.110 |
| Ovarian cancer | 3 | 1.0000302(0.9998955-1.000165) | 0.661 | 0.9999897(0.9998068-1.000173) | 0.912 | 0.9997121(0.9994227-1.000002) | 0.302 |
| Endometrial cancer | 4 | 1.000329(0.9925985-1.008120) | 0.934 | 1.002520(0.9924106-1.012733) | 0.626 | 1.006137(0.9878408-1.024771) | 0.581 |
| Head and neck cancer | 3 | 1.0000274(0.9999587-1.000096) | 0.435 | 1.0000383(0.9999576-1.000119) | 0.352 | 0.9999484(0.9998010-1.000096) | 0.617 |
| Oesophageal cancer | 3 | 1.000052(0.9999959-1.000108) | 0.069 | 1.000053(0.9999896-1.000116) | 0.102 | 1.000027(0.9999066-1.000148) | 0.734 |
| Oral cavity cancer | 3 | 1.0000078(0.9999687-1.000047) | 0.694 | 1.0000013(0.9999550-1.000048) | 0.956 | 0.9999721(0.9998881-1.000056) | 0.633 |

OR, odds ratio; CI, Confidence interval.
